# Trends in Overall Mortality, and Timing and Cause of Death among Extremely Preterm Infants near the Limit of Viability

**DOI:** 10.1371/journal.pone.0170220

**Published:** 2017-01-23

**Authors:** Jae Hyun Park, Yun Sil Chang, Sein Sung, So Yoon Ahn, Won Soon Park

**Affiliations:** 1 Department of Pediatrics, Dongsan Medical Center, Keimyung University School of Medicine, Daegu, South Korea; 2 Department of Pediatrics, Samsung Medical Center, Sungkyunkwan University School of Medicine, Seoul, South Korea; University of Cambridge, UNITED KINGDOM

## Abstract

**Objective:**

To investigate the trends in mortality, as well as in the timing and cause of death, among extremely preterm infants at the limit of viability, and thus to identify the clinical factors that contribute to decreased mortality.

**Methods:**

We retrospectively reviewed the medical records of 382 infants born at 23–26 weeks’ gestation; 124 of the infants were born between 2001 and 2005 (period I) and 258 were born between 2006 and 2011 (period II). We stratified the infants into two subgroups–“23–24 weeks” and “25–26 weeks”–and retrospectively analyzed the clinical characteristics and mortality in each group, as well as the timing and cause of death. Univariate and multivariate logistic regression analyses were done to identify the clinical factors associated with mortality.

**Results:**

The overall mortality rate in period II was 16.7% (43/258), which was significantly lower than that in period I (30.6%; 38/124). For overall cause of death, there were significantly fewer deaths due to sepsis (2.4% [6/258] *vs*. 8.1% [10/124], respectively) and air-leak syndrome (0.8% [2/258] *vs*. 4.8% (6/124), respectively) during period II than during period I. Among the clinical factors of time period, 1-and 5-min Apgar score, antenatal steroid identified significant by univariate analyses. 5-min Apgar score and antenatal steroid use were significantly associated with mortality in multivariate analyses.

**Conclusion:**

Improved mortality rate attributable to fewer deaths due to sepsis and air leak syndrome in the infants with 23–26 weeks’ gestation was associated with higher 5-minute Apgar score and more antenatal steroid use.

## Introduction

Recent improvements in perinatal and neonatal intensive care have resulted in improved survival in extremely preterm (EPT) infants near the limit of viability [1–7]. Nonetheless, EPT infants remain at the highest risk of neonatal and infant mortality worldwide [8,9]. Active treatment in infants born at or less than 24 weeks’ gestation varies widely between hospitals; in turn, there are broad inter-center differences in the survival of these infants [10]. As the decision for providng active treatment for these EPT infants is now usually indivisualized based on the shared-decision making by parents, statistics derived from populations including large numbers of EPT infants without active treatments might mispresent the infants’ chances of survival, and thus misguide the parents to forgo initiating active lifesaving intervention. Therefore, providing the most recent outcome data of EPT infants receiving active treatments is important to counsel families, and support their decision for providing active treatments to all these EPT infants [10]. The findings of this study that all EPT infants admitted to our hospital received active treatment also support the importance of providing accurate survival data for parental counseling. Furthermore, survival in infants near the limit of viability has been improved by active treatment policies, without a concomitant increase in morbidity among survivors [4, 11–15]. Taken together, these findings suggest that active treatments of EPT infants are more beneficial than harmful.

To understand the factors that contribute to death in EPT infants, it is important to identify the timing and specific causes of death. Historically, most EPT infants died within a few days of birth [16–18], immaturity and pulmonary conditions were their leading causes of death [19]. However, Patel et al. [20] reported a significant decrease in overall mortality among extremely preterm infants from 2000 through 2011, as well as a decrease in deaths related to immaturity and pulmonary causes. Overall, these findings suggest that immaturity itself may not be the main cause of death, even among the most immature infants at the verge of viability, and that their survival could be significantly improved by active and better perinatal and neonatal intensive care.

Recently, we noted that improved active perinatal and neonatal intensive care markedly improved survival among EPT infants born at 23–26 weeks’ receiving active treatments including intubation of all these infants in the delivery room throughout the study periods [21]. In the present retrospective observational study, we assessed temporal changes in overall mortality, as well as in timing and cause of death among EPT infants near the limit of viability. Specifically, we investigated two time periods between 2001 and 2011 in order to identify which factors contribute to the reduced mortality in the EPT infants receiving active treatments.

## Patients and Methods

Data collection was approved by the Institutional Review Board of Samsung Medical Center, who waived the requirement for informed consent in this retrospective chart review (IRB No. SMC 2016-03-121). We retrospectively reviewed the medical records of 382 preterm infants who were born at 23 and 26 weeks’ gestation and admitted to the neonatal intensive care unit (NICU) at Samsung Medical Center; 124 of the infants were born between January 1, 2001 and December 31, 2005 (period I), while 258 were born between January 1, 2006 and December 31, 2011 (period II). The study period was divided on the basis of changes in the survival rate of these EPT infants. We compared overall mortality, as well as timing and cause of death, between the two time periods. In admission, we compared the proportionate cause-specific mortality rate in terms of timing of death between the two time periods.

Mortality in the delivery room was included in the study, and all the infants were discharged alive or dead from our NICU; none of the infants was transferred to another center during the study period. Cause of death, defined as the disorder directly and immediately causing death of the infant, was determined by the direct and immediate cause of death ascertained from the death certificate, and these causes of death were adjudicated with the information obtained from the medical records. These listed disorders were not categorized, and instead any changes in the incidence of these specific causes of death during the study periods were analyzed. There was no death directly and immediately caused by major congenital anomalies in these infants. Pulmonary hypoplasia was defined as clinical findings associated with oligohydramnios from premature rupture of membrane (PROM) plus aggressive ventilator support, but not histologically confirmed. Air leak syndrome was defined as radiologic findings of extra-pulmonary air requiring chest tube insertion and drainage. Pulmonary hemorrhage was defined as presenting with bloody fluid from the endotracheal tube plus radiologic suggestion of pulmonary hemorrhage. Acute renal failure was defined as urine output of less than 0.5 mL/kg/ day for ≥24 hours combined with a serum creatinine level of ≥ 2.0mg/dL. Sepsis was defined as clinical symptoms/signs suggestive of sepsis including hypotension plus positive blood culture. BPD was defined as the need for supplemental oxygen and/or positive pressure to maintain oxygen saturation >90% at ≥ 28 postnatal days [22]. IVH was defined as grade≥3 and/or the ensuing post-hemorrhagic hydrocephalus (PHH) [23], and NEC was defined as ≥Bell stage IIb [24]. We analyzed clinical findings, namely gestational age (GA), birth weight, sex, mode of delivery, antenatal steroid use, chorioamnionitis, small for GA (SGA), oligohydramnios, PROM, pregnancy-induced hypertension, Apgar score at 1 and 5 minutes. We determined GA on the basis of the mother’s last menstrual period and the modified Ballard test. SGA was defined when the birth weight was less than 10^th^ percentile. Chorioamnionitis was confirmed by placental pathology, and PROM was identified when the duration of PROM was more than 24 hours.

### Statistical analysis

To analyze categorical variables, the chi-square test or Fisher’s exact test were used, while the Mann-Whitney U test or Student’s *t*-test were used to analyze continuous variables. Univariate logistic regression analyses in each subgroup were applied to calculate the crude odds ratios of individual variables for mortality. In order to identify the independent risk factors associated with mortality, multivariate logistic regression analysis was performed to control for the effects of confounding variables. Multivariate analysis included only variables with a *P* value < 0.05 on univariate analysis. The adjusted odds ratio (OR) and 95% confidence interval (CI) for each possible risk factor were calculated. A *P* value of <0.05 was considered statistically significant. All statistics were analyzed using SPSS version 18 (SPSS Inc., Chicago, IL, USA).

## Results

### Clinical characteristics

Table 1 shows the demographic and clinical findings of the infants in each subgroup and study period. The GA and birth weight of the infants born at 23 and 26 weeks’ gestation were not significantly different between periods I and II. However, the following factors were significantly higher during period II than during period I: Apgar score at 1 and 5 minutes, antenatal steroid use (especially in the infants born between 23 and 24 weeks’ gestation), and cesarean section rate (only among infants born between 23 and 24 weeks’ gestation). Other variables–namely including gender, SGA, and oligohydramnios–did not differ significantly between the two study periods.

**Table 1:** Demographic characteristics of infants born between 23 and 26 weeks’ gestation in 2001–2005 (Period I) and 2006–2011 (Period II).

	23–24Wk (N = 167)			25–26Wk (N = 215)			Total, 23-26Wk (N = 382)		
	Period I (N = 56)	Period II (N = 111)	*P* value	Period I (N = 68)	Period II (N = 147)	*P* value	Period I (N = 124)	Period II (N = 258)	*P* value
**Gestational age (week)**	23.7 ± 0.4	23.6 ± 0.5	0.102	25.5 ± 0.5	25.4 ± 0.5	0.631	24.7 ± 1.0	24.7 ± 1.0	0.732
**Birth weight (g)**	659 ± 110	633 ± 99	0.122	815 ± 121	800 ± 132	0.437	745 ± 140	728 ± 145	0.298
**Male, N (%)**	33 (58.9)	57 (51.4)	0.412	31 (45.6)	83 (56.5)	0.145	64 (51.6)	140 (54.3)	0.627
**Cesarean section, N (%)**	38 (62.5)	93 (87.3)	0.018	50 (73.5)	113 (76.9)	0.595	88 (71.0)	206 (79.8)	0.054
**Antenatal steroid, N (%)**	35 (62.6)	96 (87.3)	0.000	48 (71.6)	118 (80.8)	0.134	83 (67.5)	214 (83.6)	0.000
**Chorioamnionitis, N (%)**	26 (46.4)	60 (54.5)	0.322	29 (42.6)	70 (47.6)	0.496	55 (44.4)	130 (50.6)	0.254
**Small for GA, N (%)**	8 (14.3)	7 (6.3)	0.089	3 (4.5)	16 (10.9)	0.126	11 (8.9)	23 (8.9)	0.993
**PIH, N (%)**	2 (3.6)	5 (4.5)	0.776	9 (13.2)	19 (12.9)	0.950	11 (8.9)	24 (9.3)	0.891
**Oligohydroamnios, N (%)**	6 (20.7)	3 (12)	0.393	2 (20)	3 (16.7)	0.825	8 (20.5)	6 (14)	0.430
**PROM (day)**	1.5 ± 4.8	2.9 ± 6.7	0.178	2.1 ± 5.8	2.2 ± 4.9	0.913	1.8 ± 5.4	2.5 ± 5.8	0.290
**1-min Apgar score**	2.6 ± 1.3	4.4 ± 1.4	0.000	3.3 ± 1.7	5.0 ± 1.6	0.000	3.0 ± 1.5	4.7 ± 1.6	0.000
**5-min Apgar score**	6.5 ± 1.7	7.1 ± 1.7	0.000	6.5 ± 1.7	7.1 ± 1.7	0.010	6.1 ± 1.8	7.1 ± 1.5	0.000

GA, gestational age; PIH, pregnancy-induced hypertension; PROM, premature rupture of membrane.

### Trends in overall mortality

The overall mortality rate during hospitalization significantly improved from 30.6% during period I to 16.7% during period II (Table 2). However, despite significance in univariate analyses (Table 3), time period was not significantly associated with mortality in multivariate analyses (OR 0.818; 95% CI 0.447–1.497; *P* = 0.514) (Table 4).

**Table 2:** Overall mortality and timing of death in infants born between 23 and 26 weeks’ gestation in 2001–2005 (Period I) and 2006–2011 (Period II).

		23–24Wk (N = 167)			25–26Wk (N = 215)			Total, 23–26Wk (N = 382)		
		Period I (N = 56)	Period II (N = 111)	*P* value	Period I (N = 68)	Period II (N = 147)	*P* value	Period I (N = 124)	Period II (N = 258)	*P* value
**Overall Mortality, N (%)**		29 (51.8)	25 (22.5)	0.000	9 (13.2)	18 (12.2)	0.839	38 (30.6)	43 (16.7)	0.002
**Time of death, N (%)**	**Birth to 1 day**	3 (5.4)	2 (3.2)	0.203	3 (4.4)	3 (2.0)	0.326	6 (4.8)	5 (1.9)	0.112
	**2 to 7 days**	13 (23.2)	4 (3.6)	0.000	2 (2.9)	3 (2.0)	0.663	15 (12.1)	5 (1.9)	0.000
	**8 to 28 days**	6 (10.7)	8 (7.2)	0.167	3 (4.4)	7 (4.8)	0.951	9 (7.3)	15 (5.8)	0.363
	**>28 days**	7 (12.5)	13 (11.7)	0.294	1 (1.5)	5 (3.4)	0.433	8 (6.5)	18 (7.5)	0.812

**Table 3:** Unadjusted univariate analysis of clinical factors associated with mortality.

	23–24Wk (N = 167)			25–26Wk (N = 215)			Total, 23–26Wk (N = 382)		
	Odds ratio	95% CI	*P* value	Odds ratio	95% CI	*P* value	Odds ratio	95% CI	*P* value
**Period (2001–2005 vs 2006–2011)**	0.271	0.136–0.538	0.000	0.915	0.388–2.156	0.839	0.453	0.274–0.748	0.002
**1-min Apgar score**	0.662	0.525–0.835	0.001	0.748	0.597–0.938	0.012	0.683	0.585–0.797	0.000
**5-min Apgar score**	0.699	0.567–0.862	0.001	0.694	0.575–0.838	0.000	0.690	0.601–0.793	0.000
**Cesarean section**	0.806	0.372–1.748	0.585	4.529	1.035–19.819	0.045	1.409	0.758–2.617	0.278
**Antenatal steroid**	0.358	0.166–0.770	0.009	0.349	0.149–0.816	0.015	0.388	0.225–0.667	0.001

**Table 4:** Multivariate logistic regression analysis of clinical factors associated with mortality.

	23-24Wk (N = 167)			25-26Wk (N = 215)			Total, 23-26Wk (N = 382)		
	Odds ratio	95% CI	*P* value	Odds ratio	95% CI	*P* value	Odds ratio	95% CI	*P* value
**Period (2001–2005 vs 2006–2011)**	0.451	0.199–1.024	0.057	1.300	0.544–4.272	0.423	0.818	0.447–1.497	0.514
**1-min Apgar score**	0.593	0.627–1.271	0.530	0.897	0.662–1.214	0.480	0.855	0.691–1.057	0.148
**5-min Apgar score**	0.861	0.636–1.167	0.335	0.733	0.577–0.929	0.010	0.790	0.661–0.945	0.010
**Antenatal steroid**	0.519	0.262–1.467	0.276	0.373	0.152–0.919	0.032	0.536	0.298–0.965	0.037

### Trends in timing of death

With regards to timing of death, during period I, mortality was the common from postnatal day P2 to P7 (12.1%), while during period II, mortality was most common after P28 (7.5%) (Table 2). From P2 to P7, the mortality rate during period II had significantly improved over the period I rate (1.9% vs. 12.1%, respectively). In the subgroup analyses, the mortality rate from P2 to P7 among infants born between 23 to 24 weeks’ gestation had significantly decreased from 23.2% during period I to 3.6% during period II.

### Trends in cause-specific death

Table 5 shows the cause-specific mortality rate among the infants in each subgroup and period. The most common overall cause of death during period I was sepsis (8.1%) followed by air-leak syndrome (4.8%) and bronchopulmonary dysplasia (BPD; 4.8%), while during period II, the most common overall cause of death was intraventricular hemorrhage (IVH) and/or the ensuing post-hemorrhagic hydrocephalus (3.5%), followed by BPD (2.7%). While the overall mortality rate due to BPD had not significantly improved, the mortality rates due to sepsis and air-leak syndrome (8.1% and 4.8%, respectively, during period I) had significantly decreased to 2.4% and 0.8%, respectively, during period II. In the subgroup analyses, the mortality rate due to sepsis among the infants born between 23 and 24 weeks’ gestation had significantly decreased from, 16.1% during period I to 4.5% during period II.

**Table 5:** Causes of Death in infants born between 23 and 26 weeks’ gestation in 2001–2005 (Period I) and 2006–2011 (Period II).

Cause of Death, N (%)	23–24Wk (N = 167)			25–26Wk (N = 215)			Total, 23–26Wk (N = 382)		
	Period I (N = 56)	Period II (N = 111)	*P* value	Period I (N = 68)	Period II (N = 147)	*P* value	Period I (N = 124)	Period II (N = 258)	*P* value
**Mortality**	29 (51.8)	25 (22.5)	0.000	9 (13.2)	18 (12.2)	0.839	38 (30.6)	43 (16.7)	0.002
**Sepsis**	9 (16.1)	5 (4.5)	0.011	1 (1.5)	1 (0.7)	0.575	10 (8.1)	6 (2.4)	0.009
**Bronchopulmonary dysplasia**	6 (10.7)	6 (5.4)	0.210	0	1 (0.7)	0.495	6 (4.8)	7 (2.7)	0.283
**Intraventricular hemorrhage**	4 (7.1)	4 (3.6)	0.312	0	5 (3.4)	0.311	4 (3.2)	9 (3.5)	0.895
**Necrotizing enterocolitis**	1 (1.8)	2 (1.8)	0.994	2 (2.9)	4 (2.7)	0.927	3 (2.4)	6 (2.3)	0.955
**Air leak syndrome**	3 (5.4)	1 (0.9)	0.075	3 (4.4)	1 (0.7)	0.060	6 (4.8)	2 (0.8)	0.009
**Pulmonary hemorrhage**	2 (3.6)	2 (1.8)	0.480	1 (1.5)	3 (2.0)	0.774	3 (2.4)	5 (1.9)	0.758
**Pulmonary hypoplasia**	2 (3.6)	3 (2.7)	0.756	1 (1.5)	0	0.141	3 (2.4)	3 (1.2)	0.355

### Trends in proportionate cause-specific mortality in terms of timing of death

Table 6 shows the proportionate cause-specific mortality in terms of timing of death. The proportionate mortality rate from P2 to P7 during period I was 39.5%, which had significantly improved to 11.6% during period II. The most common cause of death between P2 and P7 during period I was sepsis, with a proportionate mortality rate of 13.2%, which had significantly improved to 0% during period II. Regarding the subgroup analyses, the proportionate mortality rate due to sepsis had only significantly improved among the infants who were born between 23 and 24 weeks’ gestation, from 17.2% during period I to 0% during period II.

**Table 6:** Proportionate cause-specific mortality rate in terms of timing of death in infants born between 23 and 26 weeks’ gestation in 2001–2005 (Period I) and 2006–2011 (Period II).

Timing of death	Cause of Death, N (%)	23–24Wk (N = 54)			25–26Wk (N = 27)			Total, 23–26Wk (N = 81)		
		Period I (N = 29)	Period II (N = 25)	*P* value	Period I (N = 9)	Period II (N = 18)	*P* value	Period I (N = 38)	Period II (N = 43)	*P* value
**Birth to 1 day**	**Mortality**	3 (10.3)	2 (8.0)	0.767	3 (33.3)	3 (16.7)	0.326	6 (15.8)	5 (11.6)	0.585
	**Air leak syndrome**	2 (6.9)	1 (4.0)	0.643	1 (11.1)	1 (5.6)	0.603	3 (7.9)	2 (4.7)	0.545
	**Pulmonary hypoplasia**	1 (3.4)	1 (4.0)	0.915	1 (11.1)	0	0.150	2 (5.3)	1 (2.3)	0.485
	**Intraventricular hemorrhage**	0	0	.	0	2 (11.1)	0.299	0	2 (4.7)	0.178
**2 to 7 days**	**Mortality**	13 (44.8)	2 (8.0)	0.003	2 (22.2)	3 (16.7)	0.726	15 (39.5)	5 (11.6)	0.004
	**Sepsis**	5 (17.2)	0	0.029	0	0	.	5 (13.2)	0	0.014
	**Intraventricular hemorrhage**	3 (10.3)	1 (4.0)	0.375	0	0	.	3 (7.9)	1 (2.3)	0.248
	**Pulmonary hemorrhage**	1 (3.4)	0	0.349	1 (11.1)	3 (16.7)	0.702	2 (5.3)	3 (7.0)	0.749
	**Pulmonary hypoplasia**	1 (3.4)	1 (4.0)	0.915	0	0	.	1 (2.6)	1 (2.3)	0.929
	**Air leak syndrome**	1 (3.4)	0	0.349	0	0	.	1 (2.6)	0	0.284
**8 to 28 days**	**Mortality**	6 (20.7)	8 (32.0)	0.344	3 (33.3)	7 (38.9)	0.778	9 (23.7)	15 (34.9)	0.271
	**Necrotizing enterocolitis**	1 (3.4)	1 (4.0)	0.915	1 (11.1)	4 (22.2)	0.484	2 (5.3)	5 (11.6)	0.309
	**Sepsis**	3 (10.3)	3 (12.0)	0.847	0	0	.	3 (7.9)	3 (7.0)	0.875
	**Pulmonary hemorrhage**	1 (3.4)	2 (8.0)	0.467	0	0	.	1 (2.6)	2 (4.7)	0.631
	**Air leak syndrome**	0	0	.	2 (22.2)	0	0.038	2 (5.3)	0	0.128
	**Acute renal failure**	0	0	.	0	2 (11.1)	0.299	0	2 (4.7)	0.178
**>28 days**	**Mortality**	7 (24.1)	13 (52.0)	0.035	1 (11.1)	5 (27.8)	0.326	8 (21.1)	18 (41.9)	0.045
	**Bronchopulmonary dysplasia**	6 (20.7)	6 (24.0)	0.770	0	1 (5.6)	0.471	6 (15.8)	7 (16.3)	0.952
	**Sepsis**	1 (3.4)	2 (8.0)	0.467	1 (11.1)	1 (5.6)	0.603	2 (5.3)	3 (7.0)	0.749
	**Intraventricular hemorrhage**	0	2 (8.0)	0.121	0	3 (16.7)	0.194	0	5 (11.6)	0.030
	**Necrotizing enterocolitis**	0	1 (4.0)	0.277	0	0	.	0	1 (2.3)	0.344

From P8 and P28, the proportionate mortality rate was 23.7% during period, which had increased during period II, without statistical significance, to 34.9%. In the subgroup analyses, from P8 to P28, the proportionate mortality rate due to air-leak syndrome in the infants born between 25 and 26 weeks’ gestation during period I was 22.2%; this had significantly improved (to 0%) during period II.

During period I, the overall proportionate mortality rate after P28 was of 21.1%; this had significantly increased (to 41.9%) during period II. The mortality due to the most common cause of death after P28, BPD, did not change significantly between the study periods; however, mortality after P28 due to the sequelae of IVH such as PHH had significantly increased from 0% during period I to 11.6% during period II.

### Clinical factors associated with mortality

In the univariate analyses of possible clinical factors associated with mortality, the following were identified as significant: study period, 1- and 5-minute Apgar score, antenatal steroid use (Table 3). However, in multivariate analyses, only 5-minute Apgar score (OR: 0.790, 95% CI: 0.661–0.945) and antenatal steroid use (OR: 0.536, 95% CI: 0.298–0.965) were significantly associated with decreased mortality (Table 4).

## Discussion

Mortality rates in EPT infants have been declining incrementally during the past decades due to continuing improvements in perinatal and neonatal intensive care [1–7]. However, it remains uncertain whether survival rate has improved beyond 23–24 weeks’ gestation [9, 25]. Recently, several studies reported that active lifesaving treatments improved mortality rates in EPT infants at the known verge of viability [4, 11–15]. Indeed, inter-center differences in the mortality of EPT infants at the verge of viability are primarily due to variations in the active perinatal and neonatal treatment of these most immature infants [10]. In the present study, while active treatments were applied to all EPT infants throughout the study periods, we observed significantly much more improved mortality rates–from 51.8% to 22.5% during last decade–in EPT infants, especially in those who were born between 23 to 24 weeks’ gestation. Therefore, our current data about the mortality rate in these infants might inform guidelines for the care of these most immature infants, as well as improve the counseling that families receive regarding the infants’ survival [1, 26]. Furthermore, provision of better perinatal and neonatal intensive care significantly improved 5 min Apgar score and reduced sepsis and air leak syndrome besides active life saving treatments would also much more improve the mortality of the EPT infants. Taken together, these findings suggest that the limit of viability in EPT infants is not static, and that it could be much more improved not only with active treatments but also with better perinatal and neonatal intensive care [4].

EPT birth is a leading cause of infant mortality and morbidity in survivors; for this reason, researchers are concerned that improved mortality rates in EPT infants at the verge of viability will be accompanied by an increase in disabling morbidities among survivors [5, 27, 28]. However, in the present and other studies [3, 6, 7, 11, 15, 29], the proportion of major morbidities among survivors was unchanged, or even reduced, despite their improved viability. Moreover, the improved survival in EPT infants between 23 and 24 weeks’ gestation as a result of active perinatal and neonatal treatments in the current study was associated with our previous finding of significantly reduced prevalence of morbidities, such as BPD and nosocomial sepsis, in more mature infants born between 25 and 26 weeks’ gestation [21]. In concordance with our data, Stoll et al. [2] reported most markedly improved survival during the last two decades among infants born between 23 and 24 weeks’ gestation, as well as significantly improved survival, without major morbidity, in infants born between 25 and 28 weeks’ gestation. Overall, these findings suggest that the aforementioned active perinatal and neonatal managements are associated with significantly increased survival rates, without major morbidities in the more mature EPT infants. As a result, considerably more EPT infants enjoy a potentially good outcome [30].

Several recent studies reported improved survival among infants near the limit of viability [1, 2, 11, 15]; however, only one study evaluated the timing and cause-specific mortality associated with this reduction [20]. In a study of Patel et al. [20], the number of deaths per 1000 live births among infants born between 22 and 28 weeks’ gestation had significantly improved–from 275 to 258. Most notably, the mortality rate decreased among infants born at 23 weeks’ gestation–from 753 and 431 per 1,000 births–between the 2000–2003 and 2008–2011 periods, while the mortality rate among infants born at 24 weeks’ gestation decreased from 691 to 390 per 1,000 births. However, the timing of death was not significantly different among the study periods, with 40.4% of deaths occurring within 12 hours of birth. Mortalities during this period were most commonly attributed to immaturity, and these infants were mostly not intubated. In contrast, in the present study, overall mortality in the infants born between 23 and 26 weeks’ gestation had significantly improved–from 30.6% to 16.7%–especially among infants born between 23 and 24 weeks’ gestation, wherein it had reduced from 51.8% to 22.5% between periods I and II. However, during both study periods, the proportion of death occurring on P0 or P1 was 13.6%. Overall, these findings suggest that active perinatal and neonatal treatments including antenatal steroid use, cesarean section, and delivery room intubation may prevent early death and thus improve survival among EPT infants–even those near the limit of viability [19].

It is important that clinicians and researchers identifying the practices primarily responsible for the improved mortality rates in these infants. Patel et al. [20] conjectured that mortality was improved because there fewer pulmonary deaths, for instance, from respiratory distress syndrome or BPD. They also noted that fewer deaths occurred from infection or brain injuries. In the present study, mortality was improved mainly because there were fewer deaths from sepsis and air-leak syndrome. This suggests that survival in EPT infants at the verge of viability could be improved by identifying and applying best key clinical care strategies, including gentle meticulous respiratory care, as well as prevention of nosocomial sepsis [31–36]. However, since chronic diseases such as BPD and severe IVH have become the major cause of death in the present study, it will be necessary to develop new therapeutic modalities, such as stem cell therapy, to overcome the mortality caused by these intractable diseases [37, 38].

In the present study, during period II, there were significantly higher rates of delivery by cesarean section and antenatal steroid use, especially among infants born between 23 and 24 weeks’ gestation; furthermore, the 1- and 5-minute Apgar scores were better during period II. However, only the clinical factors of 5-minute Apgar score and antenatal steroid uses were significantly associated with mortality in multivariate analyses. These findings suggest that both active antenatal steroid use [4, 11, 25, 27, 39, 40] and universal active delivery room resuscitation could improve the mortality rate, and that individual treatment decisions regarding EPT infants near the limit of viability should not be made in the delivery room [41]. Further studies would be necessary to identify the causal relationship between these identified clinical factors by multivariate analyses and mortality in the EPT infants near the limit of viability.

The present study had several limitations; for instance, it had a retrospective design. Furthermore, even though we reviewed all the medical records to confirm the primary cause of death written on death certificate, it is sometimes difficult to differentiate a single primary cause of death in a complex situation where multiple causes interact; for this reason, the listed cause of death may have been subjective. Another limitation was that, as our data were obtained from a single institution, the findings observed in the present study might not be generalizable to other institutions. However, we used a relatively large sample of 382 infants born between 23 and 26 weeks’ gestation at a single institution; moreover, the infants had similar baseline characteristics, which implies that this study was better placed to identify those therapeutic strategies that are associated with improved mortality rates in these infants. The relatively small sample size of the subgroups might be another limitation of this study. However, although consistency of treatment have not been measured, treatments given by the same neonatologists in a single center and statistically significant improved mortality even in these small sample size subgroups night redeem this limitation.

In conclusion, overall mortality, especially in infants born between 23 and 24 weeks’ gestation, was significantly decreased, as were deaths due to sepsis and air-leak syndrome. The proportion of deaths caused by complications of severe IVH increased. The improved mortality in these EPT infants was significantly associated with higher 5-minute Apgar score and more antenatal steroid use. These findings underscore the developing and implementing new therapeutic strategies to overcome the currently known limit of viability in EPT infants.
